# Altitude and its inverse association with abdominal obesity in an Andean country: a cross-sectional study

**DOI:** 10.12688/f1000research.20707.2

**Published:** 2019-12-27

**Authors:** Jaime Pajuelo-Ramírez, Harold Torres-Aparcana, Rosa Agüero-Zamora, Antonio M. Quispe

**Affiliations:** 1 Universidad Nacional Mayor de San Marcos, Lima, Peru; 2 Clínica San Felipe, Lima, Peru; 3 Universidad San Martin de Porres, Lima, Peru; 4 Hospital Nacional Dos de Mayo, Lima, Peru; 5 Universidad Continental, Huancayo, Peru

**Keywords:** Abdominal obesity, Altitude, Obesity, Waist Circumference, Peru

## Abstract

**Background**: Abdominal obesity represents an accurate predictor of overall morbidity and mortality, which is worrisome because it is also continuously increasing across Andean countries. However, its relationship with altitude remains unclear. The objective of this study was to assess the association between altitude and abdominal obesity in Peru, and how sociodemographic variables impact this association.

**Methods**: We estimated the prevalence of abdominal obesity in Peru and analyzed its association with altitude using the data from the 2012-2013 National Household Survey (ENAHO). During this survey, a representative sample of Peruvians was screened for abdominal obesity, using waist circumference as a proxy, and the Adult Treatment Panel III guidelines cutoffs.

**Results**: Data were analyzed from a sample of 20 489 Peruvians (51% male). The prevalence of abdominal obesity was estimated at 33.6% (95% CI: 32.5 to 34.6%). In Peru, altitude was significantly and inversely associated with abdominal obesity, decreasing with higher altitudes: 1500-2999 meters above mean sea level (MAMSL) vs <1500 MAMSL, adjusted prevalence rate [aPR]= 0.90 (95% CI: 0.84 to 0.96); ≥3000 MAMSL vs <1500 MAMSL, aPR= 0.78 (95% CI: 0.72 to 0.84), when adjusting by age, gender and residence area (rural/urban). However, this association was significantly modified by age and gender ( *p*< 0.001).

**Conclusion**: Abdominal obesity is highly prevalent in Peru and decreases significantly with altitude, but age and gender modify this association. Thus, abdominal obesity appears to affect older women from low altitudes more than younger men from high altitudes.

## Introduction

The increasing prevalence of obesity represents a significant public health problem across low- to high-income countries ^1^. The main reason is that obesity is strongly associated with morbidity and mortality, mostly due to type 2 diabetes, cancer and cardiovascular diseases ^2^. However, body fat distribution, particularly that of abdominal obesity, has been reported as a better predictor of overall morbidity and mortality than total adiposity or obesity defined by body mass index (BMI) ^3, 4^. Furthermore, abdominal obesity is difficult to diagnose in routine clinical care because it requires access to computed tomography ^5^ or magnetic resonance imaging ^6^ for precise quantification. Anthropometric measures of abdominal obesity include waist circumference, waist-to-height ratio, waist-to-hip ratio, and the conicity index ^7, 8^. Thus, the most commonly used surrogate to diagnose abdominal obesity in clinical care and research examinations is waist circumference ^9, 10^.

In Peru, as in most Latin-American countries, the prevalence of obesity among children, adolescents and adults have grown consistently in recent decades. Among Peruvian adults, estimates of the national prevalence of obesity have grown from approximately 9% in 1975 to 21% in 2017 ^11^. However, this prevalence seems to vary substantially by altitude ^12^.

Epidemiological studies carried out in the United States ^13^ and Peru ^12^ among adults and children ^14^ have described an inverse association between altitude and obesity. A previous study reported that the prevalence of obesity in Peru decreases by approximately 26% at between 1500–2999 meters above mean sea level (MAMSL), and by 46% at over 3000 MAMSL, as compared to at 0–499 MAMSL ^12^.

Consequently, this study further assesses the association between altitude and abdominal obesity, when adjusted by standard sociodemographic variables. Additionally, we plan to estimate the prevalence of abdominal obesity by different cutoffs.

## Methods

### Study design

The study employed a cross-sectional multistage study design. Data were accessed from the Peruvian National Household Survey (ENAHO), undertaken annually by the Peruvian National Institute of Statistics and Information (INEI) and the National Center for Food and Nutrition (CENAN) to assess social living conditions. For this purpose, the INEI and CENAN surveyed a representative sample of the Peruvian population using a probabilistic, stratified, multi-stage design, independent for each region, to collect data on participants of ≥2 months of age ^15^. Briefly, the ENAHO sample household residents from all regions of Peru (third sampling level), sampling clusters of one or more blocks of ~120 houses (second sampling level) and sampling cities with 2000 or more inhabitants in the urban area or 500–2000 inhabitants in the rural area (first sampling level). ENAHO survey eligibility criteria were Peruvian households inhabitants, including family members, non-family members and domestic workers (with or without payment) that cohabitated during the 30 days prior to the survey, excluding pensions of 10 or more inhabitants ^15^. In this study, we used ENAHO 2013 data to assess the prevalence of abdominal obesity and its association with altitude, while adjusting for their primary demographics and design effect. Out of 45 164 observations, people aged 20 years or older were included. We excluded pregnant women, and those observations with unreliable data.

### Variables of interest

The study outcome was abdominal obesity: we used waist circumference (WC) as a proxy for its diagnosis. During the ENAHO survey, trained personnel measured the subject’s WC at the vertical position of the midpoint between the lowest rib and the border of the iliac crest ^10^. We interpreted this measurement by using the cutoffs proposed by Adult Treatment Panel III guidelines (ATP III) for abdominal obesity: WC >102 cm for men and >88 cm for women ^16, 17^. Additionally, for comparison, we assessed the cutoffs proposed by the Latin-American Diabetes Association (ALAD): WC ≥94 cm for men and ≥88 cm for women ^18^ and that of the International Diabetes Federation (IDF): WC >90 cm for men and >80 cm for women ^19^. Furthermore, we define abdominal obesity as a weight to height ratio (WtHR) ≥0.5 ^20^ and obesity as a BMI ≥30 kg/m ^2^. For this purpose, WHtR was defined as subject’s waist circumference divided by their height, both measured in cms.; and BMI was defined as the body weight (kg) divided by the square of the body height (m ^2^).

To facilitate comparisons and interpretability, we categorized altitude (measured by GPS) as low (<1500 MAMSL), moderate (1500–2999 MAMSL), and high (≥3000 MAMSL). Likewise, individuals were categorized by age as young adults (20–39 years), adults (40–59 years) and elders (≥60 years). We classified the area of residence as rural using ENAHO/INEI standard definition, which define an area as rural or rural town, if has no more than 100 contiguous households grouped or have more than 100 households scattered or disseminated without forming blocks or cores ^15^. Nutritional status was assessed by BMI and categorized using WHO standard cutoffs as underweight (<18.5 kg/m ^2^), normal (18.5–24.9 kg/m ^2^), overweight (25–29.9 kg/m ^2^), and obese (≥30 kg/m ^2^) ^21^.

### Statistical analysis

We estimated the prevalence of abdominal obesity by considering survey sampling weights by using STATA survey (svy) commands and excluding registers with missing study outcomes. We assessed bivariate correlation by estimating the Spearman’s rank-order correlation coefficient. Considering that the prevalence of abdominal obesity in Peru is not rare ^11^, we estimated the adjusted prevalence ratio as a measure of association instead of the odds ratio ^22^. Thus, we used a log-binomial regression model that has robust variance, rather than a Poisson regression model, to adjust our prevalence ratio estimates by gender, age group and area of residence ^23^. Finally, we tested for interaction between gender and altitude, and between age and altitude using the Wald test because of the consensus that obesity prevalence vary by gender and age ^3^. All statistical analyses were performed using STATA/MP 14.0 for Mac (StataCorp LP, College Station, TX), and the results of statistical tests were interpreted and summarized with 95% confidence intervals.

### Ethical statement

According to the Regulation of Ethics in Research of the Peruvian National Institute of Health, this study did not require approval or exemption from an ethics committee because the database is publicly available. Study dataset was published using Figshare, which requested to hide subjects’ age and to strictly limit the data availability to only the variables analyzed in this study.

## Results

### Characteristics of the study population

We analyzed a population sample of 20 489 subjects from 703 different locations across 25 administrative regions of Peru. To summarize population demographics, most subjects were either female (51.6%), adults between 20 to 39 years of age (39.8%), or inhabitants from urban areas (79.6%) ^24^. Of these three demographic measures, both age groups ( *p*=0.0006) and area of residence ( *p*<0.0001) distribution varied significantly by altitude ( Table 1).

**Table 1.** Peru’s demographics by altitude level.

Characteristics	Number of participants (%)			Total
	<1500 MAMSL	1500–2999 MAMSL	≥3000 MAMSL	
**Age (years)**				
20 to 39	5092 (40.7)	1262 (40.0)	1649 (36.4)	8003 (39.8)
40 to 59	4804 (37.8)	1226 (36.8)	1795 (38.0)	7825 (37.7)
≥60	2555 (21.5)	818 (23.2)	1288 (25.6)	4661 (22.5)
**Gender**				
Female	6796 (51.4)	1848 (52.0)	2703 (52.4)	11347 (51.6)
Male	5655 (48.6)	1458 (47.6)	2029 (47.6)	9142 (48.4)
**Residence area**				
Urban	9861 (89.4)	1744 (61.7)	2354 (57.3)	13959 (79.6)
Rural	2590 (10.6)	1562 (38.3)	2378 (42.7)	6530 (20.4)
**Body Mass Index**				
Underweight	139 (1.2)	46 (1.4)	93 (1.8)	278 (1.3)
Normal	4257 (33.1)	1614 (46.4)	2560 (52.1)	8430 (38.5)
Overweight	5317 (42.8)	1173 (37.2)	1578 (34.3)	8068 (40.5)
Obesity	2738 (22.9)	473 (15.0)	501 (11.8)	3712 (19.7)
**Abdominal obesity**				
By Waist ATP III	4402 (37.1)	893 (28.7)	1098 (24.3)	6393 (33.6)
By Waist ALAD	5683 (49.4)	1107 (37.0)	1394 (31.9)	8184 (44.4)
By Waist IDF	8330 (69.1)	1804 (57.2)	2342 (51.0)	12476 (64.1)
By WtHR >0.5	10632 (86.1)	2629 (80.8)	3666 (77.9)	16903 (83.9)

Parameters estimated considering the design effect and the complexities of the survey; WtHR, waist-to-height ratio; MAMSL, meters above mean sea level; ATP III, Adult Treatment Panel III guidelines; ALAD, Latin-American Diabetes Association; IDF, International Diabetes Federation.

### Prevalence of obesity and abdominal obesity in Peru

**Figure 1.**
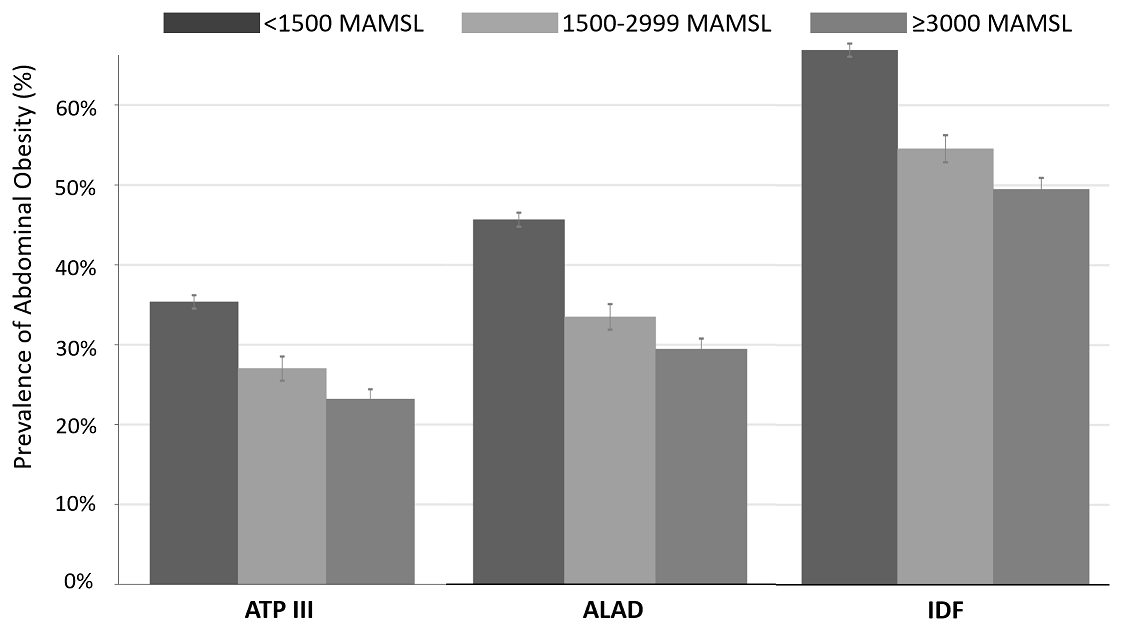
Prevalence of abdominal obesity by altitude and by different cutoffs. ATP III, Adult Treatment Panel III guidelines; ALAD, Latin-American Diabetes Association; IDF, International Diabetes Federation; MAMSL, meters above mean sea level.

The prevalence of abdominal obesity in Peru was 33.6% (95% CI: 32.5% - 34.6%) when using WC and the ATP III cutoff, 44.4% (95% CI: 43.2% - 45.6%) using the ALAD cutoff and 64.1% (95% CI: 63.0% - 65.2%) using the IDF cutoff ( Table 1). Regardless of the cutoff used (i.e. ATP III, ALAD, or IDF), the prevalence of abdominal obesity decreased significantly ( *p*<0.001) with altitude: abdominal obesity was more prevalent at low elevations (<1500 MAMSL), less prevalent at moderate elevations (1500–2999 MAMSL), and lowest at high elevations (≥3000 MAMSL) ( Table 1 and Figure 1). 

Similarly, we estimated the prevalence of abdominal obesity in Peru using waist to height ratio (WtHR) to be 83.9% (95% CI: 83.1%-84.6%). Like the previous model that employed WC as a surrogate measure of abdominal obesity, the present model also demonstrated an inverse association between abdominal obesity and altitude category. In this model, the prevalence of abdominal obesity (as defined by WtHR) was 86.1% (95% CI: 85.1%-87.1%) for those at low altitudes, 80.7% (95% CI: 78.9%-82.7%) at moderate altitudes, and 77.9% (95% CI: 76.1% to 79.6%) at high altitudes ( *p*<0.001) ( Table 1).

We estimated the total prevalence of obesity in Peru by BMI to be 19.7% (95% CI: 18.9%-20.6%). Like that of abdominal obesity, the prevalence of obesity was inversely related to the categories of altitude that we defined. Obesity prevalence was 22.9% (95% CI: 21.7%-24.1%) at low elevations, 15.0% (95% CI: 13.5%-16.6%) at moderate elevations, and 11.8% (95% CI: 10.6%-13.1%) at high elevations for those living at or over 3000 MAMSL, respectively ( *p*<0.001) ( Table 1).

### Variability of abdominal obesity by different cutoffs in Peru

Estimates of abdominal obesity prevalence vary significantly with altitude and in models that use different standard diagnostic cutoffs. When comparing the estimated prevalence of abdominal obesity using ATP III, ALAD and IDF cutoffs ( Table 1 and Figure 1), there were significant differences between them ( *p*<0.001 at each paired comparison). The same variability was observed regardless of age group, gender, and residence area ( Table 2). Furthermore, in the correlation analysis ( Table 3), we found that using the ATP III cutoff resulted in a stronger correlation with obesity by BMI (Spearman´s ρ = 0.55; *p*<0.001), as compared with the ALAD (Spearman´s ρ = 0.53; *p*<0.001) and IDF cutoffs (Spearman´s ρ = 0.37; *p*<0.001). However, the ATP III cutoff also has a weaker correlation with altitude (Spearman´s ρ = 0.12; *p*<0.001). Additionally, we found that the prevalence of abdominal obesity, as defined by WtHR >0.5, has only a moderate correlation with the prevalence of obesity by BMI (Spearman´s ρ = 0.43; *p*<0.001) and a weak correlation with altitude ( Table 3).

**Table 2.** Abdominal obesity prevalence by demographic and nutritional factors at different altitude levels.

	ATP III				ALAD				IDF			
	<1500 MAMSL % (95% CI)	1500– 2999 MAMSL % (95% CI)	≥3000 MAMSL % (95% CI)	Total % (95% CI)	< 1500 MAMSL % (95% CI)	1500– 2999 MAMSL % (95% CI)	≥3000 MAMSL % (95% CI)	Total % (95% CI)	<1500 MAMSL % (95% CI)	1500– 2999 MAMSL % (95% CI)	≥3000 MAMSL % (95% CI)	Total % (95% CI)
**All**	37	29	24	34	49	37	32	44	69	57	51	64
	(36-38)	(27-30)	(22-26)	(33-35)	(48-51)	(35-40)	(30-34)	(43-46)	(68-71)	(55-60)	(49-53)	(63-65)
**Age** **groups**												
20 to 39 years	25	19	20	24	35	25	24	32	56	46	45	53
	(24-27)	(16-22)	(17-22)	(22-25)	(33-37)	(22-28)	(22-27)	(30-33)	(54-58)	(42-49)	(42-48)	(51-54)
40 to 59 years	44	38	31	41	58	48	40	54	78	68	58	73
	(42-47)	(34-41)	(27-34)	(39-42)	(56-61)	(44-52)	(37-44)	(52-55)	(76-80)	(64-71)	(54-62)	(71-74)
≥60 years	47	31	22	39	61	41	30	52	79	60	49	70
	(44-50)	(27-36)	(19-25)	(37-41)	(59-64)	(35-46)	(27-34)	(50-54)	(77-81)	(55-64)	(45-52)	(68-71)
**Gender**												
Female	56	44	40	51	56	44	40	51	80	71	66	76
	(54-58)	(41-47)	(38-43)	(50-53)	(54-58)	(41-47)	(38-43)	(50-52)	(79-82)	(69-74)	(63-68)	(75-77)
Male	18	12	7	15	43	29	23	37	57	42	36	51
	(16-19)	(10-15)	(5-8)	(14-16)	(40-45)	(26-32)	(26-38)	(35-39)	(55-59)	(38-45)	(31-38)	(49-53)
**Residence** **area**												
Urban	39	35	31	37	51	45	42	49	71	66	61	69
	(37-40)	(32-37)	(29-34)	(36-39)	(50-53)	(43-48)	(39-45)	(48-51)	(70-72)	(63-68)	(58-64)	(68-70)
Rural	23	19	15	18	32	23	19	25	53	43	37	44
	(20-25)	(16-22)	(13-17)	(17-20)	(29-36)	(21-26)	(16-21)	(23-27)	(40-56)	(40-47)	(34-41)	(42-47)

Estimates considering the design effect and the complexities of the survey; abdominal obesity estimated using the cutoffs proposed by Adult Treatment Panel III guidelines (ATP III); MAMSL, meters above mean sea level; ALAD, Latin-American Diabetes Association; IDF, International Diabetes Federation.

**Table 3.** Correlation between altitude and each of our parameters of interest.

	Altitude	BMI	Waist	WtHR	AO-ATP III	AO-ALAD	AO-IDF	AO-WtHR >0.5
BMI	-0.1872							
Waist	-0.2049	0.8406						
WtHR	-0.1437	0.8375	0.8997					
AO-ATP III	-0.1241	0.6396	0.6573	0.7264				
AO-ALAD	-0.1559	0.6823	0.7680	0.7539	0.8258			
AO-IDF	-0.1608	0.6175	0.6861	0.7067	0.5397	0.6536		
AO-WtHR >0.5	-0.1218	0.6349	0.7462	0.7552	0.5613	0.7007	0.6132	
Obesity	-0.1350	0.7438	0.6137	0.6271	0.5546	0.5330	0.3679	0.4340

All the correlations estimated in the table resulted in a p-value <0.001 when tested as equal to zero. BMI, body mass index; AO, abdominal obesity; WtHR, waist to height ratio; ATP III, Adult Treatment Panel III guidelines; ALAD, Latin-American Diabetes Association; IDF, International Diabetes Federation.

### Variability of abdominal obesity by altitude in Peru

The prevalence of abdominal obesity and obesity vary significantly by altitude in Peru and are inversely associated with altitude category (trend analysis *p*<0.001 for both), regardless of age group, gender and residence area ( Table 4). Both abdominal obesity and obesity prevalence were significantly higher among females than males ( *p*<0.001 for both) and across urban areas than in rural areas ( *p*<0.001 for both). The prevalence of obesity and abdominal obesity were significantly lower among young adults (20–39 years) than among adults (40–59 years); however, both obesity and abdominal obesity prevalence were significantly higher in young adults than elders (≥60 years old).

**Table 4.** Obesity and abdominal obesity prevalence by demographic at different altitudes.

Characteristics	Abdominal obesity * ^+^				Obesity by BMI *			
	Prevalence (95% CI)				Prevalence (95% CI)			
	<1500 MAMSL	1500–2999 MAMSL	≥3000 MAMSL	Total	<1500 MAMSL	1500–2999 MAMSL	≥3000 MAMSL	Total
**All**	37	29	24	36	23	15	12	20
	(36-39)	(26-31)	(22-26)	(33-35)	(21-24)	(14-17)	(11-13)	(19-21)
**Age groups**								
20 to 39 years	25	19	20	24	18	10	8	15
	(24-27)	(16-22)	(17-22)	(22-25)	(16-19)	(9-12)	(6-10)	(14-16)
40 to 59 years	44	38	30	41	27	20	17	24
	(42-47)	(34-41)	(27-34)	(39-42)	(25-29)	(18-23)	(14-19)	(23-26)
≥60 years	47	31	22	39	25	15	10	20
	(44-50)	(27-36)	(19-25)	(37-41)	(22-27)	(12-19)	(8-12)	(19-22)
**Gender**								
Female	56	44	40	51	26	20	18	23
	(54-58)	(41-47)	(38-43)	(50-53)	(24-28)	(18-22)	(16-20)	(22-25)
Male	18	12	7	15	20	10	5	16
	(16-19)	(10-15)	(5-8)	(14-16)	(18-22)	(8-12)	(4-7)	(14-17)
**Residence area**								
Urban	39	35	31	37	24	20	16	22
	(37-40)	(32-37)	(29-34)	(36-39)	(23-25)	(17-21)	(15-17)	(21-23)
Rural	23	19	15	18	13	8	6	9
	(20-25)	(16-22)	(13-17)	(17-20)	(11-15)	(7-10)	(5-7)	(8-10)

*Estimates considering the design effect and the complexities of the survey; +, abdominal obesity estimated using the cutoffs proposed by Adult Treatment Panel III guidelines (ATP III); MAMSL, meters above mean sea level; BMI, body mass index; 95% CI, confidence intervals of 95%.

### Abdominal obesity and its association with altitude in Peru

**Figure 2.**
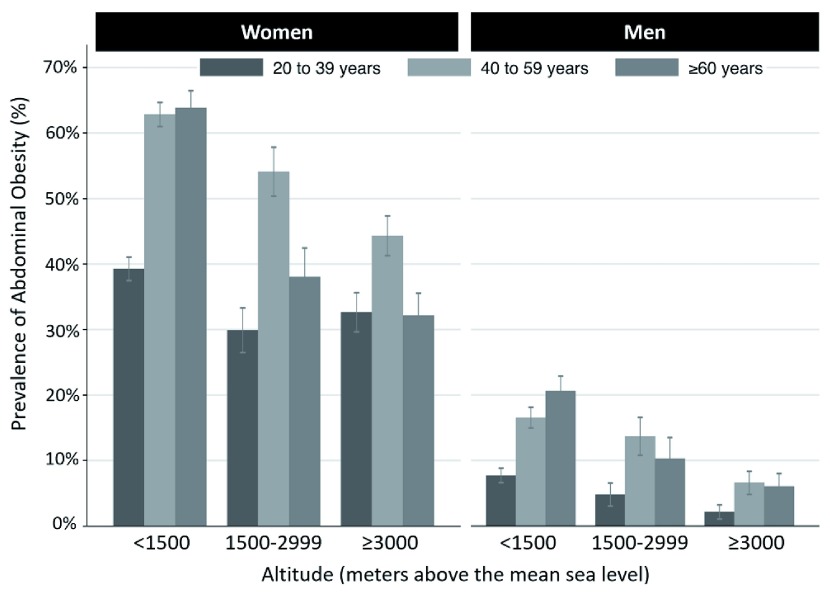
Prevalence of abdominal obesity (by Adult Treatment Panel III guidelines cutoffs) by age group and altitude. The association between abdominal obesity and altitude vary greatly by gender and age group, which behave as effect modifiers.

Regression analyses demonstrated that the prevalence of abdominal obesity was significantly associated with altitude when either unadjusted and adjusted by age groups, gender, and residence. Additionally, we observed significant effect modification of this association by age group and gender, which seems to be particularly high at altitudes over 3000 MAMSL. Once adjusted by the interaction terms, the association between abdominal obesity and altitude varies significantly by gender, age group and residence area, with different patterns of distribution at different altitudes. At lower altitudes (<1500 MAMSL), the prevalence of abdominal obesity exhibits a positive trend increasing by age group, while above 1500 MAMSL, it exhibits an inverted-u shaped relationship ( Figure 2).

### Abdominal obesity and its associated factors in Peru

In the regression analysis, we found that altitude, age groups, gender, and residential area were significantly associated with the prevalence of abdominal obesity in Peru ( Table 5). Based on our multivariate regression analysis outputs, we observed that the prevalence of abdominal obesity decreased with altitude, increased with age, and is lower among male and rural populations. However, contrary to what was observed for the prevalence of abdominal obesity by altitude in the case of gender and residence area, both of which decrease with altitude, the variability of the prevalence of abdominal obesity by age group exhibits different patterns of distribution at different altitudes. Overall, the prevalence of abdominal obesity in Peru is higher among women ≥60 years living at <1500 MAMSL (68.4%; 95% CI, 64.6 to 71.9), and lower among men between 20 to 39 years of age living al ≥3000 MAMSL (2.8%; 95% CI, 1.6 to 4.8), exhibiting an inverted-u shaped relationship ( Figure 2).

**Table 5.** Factors associated with abdominal obesity (by ATP III) in Peru.

Factors	Unadjusted PR	CI 95%	Adjusted PR * w/o IT	CI 95%	Adjusted PR ** w/ IT	CI 95%
**Altitude (MAMSL)**						
<1500	Ref.		Ref.		Ref.	
1500 to 2999	0.77	0.71 to 0.84 ^‡^	0.90	0.84 to 0.96 ^‡^	0.86	0.75 to 0.97 ^†^
≥ 3000	0.65	0.60 to 0.71 ‡	0.78	0.72 to 0.84 ^‡^	0.98	0.87 to 1.11 °
**Age group (years)**				
20–39	Ref.		Ref.		Ref.	
40–59	1.73	1.62 to 1.85 ^‡^	1.67	1.57 to 1.77 ^‡^	1.66	1.54 to 1.79 ^‡^
≥60	1.66	1.55 to 1.79 ^‡^	1.68	1.57 to 1.80 ^‡^	1.77	1.64 to 1.91 ^‡^
**Gender**						
Female	Ref.		Ref.		Ref.	
Male	0.29	0.26 to 0.32 ^‡^	0.30	0.27 to 0.33 ^‡^	0.33	0.30 to 0.36 ^‡^
**Residence area**						
Urban	Ref.		Ref.		Ref.	
Rural	0.49	0.45 to 0.53 ^‡^	0.57	0.52 to 0.62 ^‡^	0.58	0.53 to 0.63 ^‡^
**Altitude x gender**						
1500 to 2999 x Male					0.85	0.67 to 1.08 ^°^
≥3000 x Male					0.5	0.38 to 0.66 ^‡^
**Altitude x age group**						
1500 to 2999 x 40–59					1.17	1.01 to 1.36 ^†^
(1500 to 2999 x ≥60) or (≥3000 x 40 to 59)					0.95	0.83 to 1.08 °
≥3000 x ≥60					0.63	0.53 to 0.75 ^‡^

Design effect was considered according to complex survey data; ATP III, Adult Treatment Panel III guidelines; PR, prevalence rates; IT, interaction terms; CI 95%, confidence intervals of 95%, MAMSL: meters above mean sea level; *, Adjusted prevalence rate without interaction terms; **, Adjusted prevalence rate with interaction terms; °, non-significant p-value; ^†^, p-value <0.05; ^‡^, p-value <0.001.

## Discussion

The prevalence of abdominal obesity in Peru is high and decreases with altitude, an association that is modified by age and gender. This prevalence was higher among women over 60 years of age below 1500 MAMSL, and lowermost among men 20 to 39 years of age over 3000 MAMSL, exhibiting an inverted-u shaped relationship. Understanding the intricacies of this association is critical in countries with high elevation such as Peru, where approximately 20% of the Peruvian population lives at or above 3000 MAMSL ^25^. 

The usefulness of WC as an indicator of abdominal obesity is quite clear; however, there is a permanent discussion regarding the cutoffs for its diagnosis. WC varies by ethnic groups, which has generated the recommendation that each country or region produces its cutoffs ^26^. Worldwide, the most used cutoffs for WC are the ones proposed by the ATP III, which are primarily specific for adult European Caucasian populations ^16, 17^.

There are some efforts in Latin America to propose WC cutoffs for their population. A recent study carried out in five Latin American countries recommended using cutoffs of 90–92 cm for women and 94 cm for men ^27^. In Peru, the PREVENTION study proposed WC cutoffs at high altitude ( ^~^2600 MAMSL) of 87 cm for women and 97 cm for men based on abnormalities of intima-media thickness and cardiovascular manifestations ^28^. Similarly, different countries have proposed their cutoffs for WC, including Portugal (91 and 97 cm) ^29^, China (80 and 84 cm) ^30^, and South Asian countries (84 and 88 cm) ^31^. In our study, different cutoffs produced a wide range of estimates for the prevalence of abdominal obesity. We observed that when using ATP III cutoffs, the estimated prevalence of abdominal obesity was over three times higher among women than in men (51% vs 15%).

Furthermore, regardless of altitude, these differences seem to be even larger ≥3000 MAMSL (40% vs 7%). These differences are similar to those reported previously ^32^, so we believe they can be explained by both the altitude effect and the cutoffs itself, which are gender-differentiated. Further studies are needed to assess the necessity of specific cutoffs corrected by altitude, gender, and age.

Another important finding of our study is that the prevalence of abdominal obesity varies significantly between urban and rural areas, a difference that remains consistent at different altitudes. As reported elsewhere, the prevalence of abdominal obesity in Peru is higher in urban areas than in rural areas ^33^, but also shows a slower increase in time in WC compared to rural areas ^34^. However, such a difference between urban and rural areas seems to increase with higher altitudes, ranging from 1.7:1 at <1500 MAMSL to 2.1:1 at ≥3000 MAMSL. This finding is relevant in countries with large populations living over 3000 MAMSL, due to the cardiovascular risk that this could imply.

Regardless of WC cutoffs utilized, the mean WC in the Peruvian population living at high altitudes is high. In our study, at >3000 MAMSL the mean WC among men was 87.1 cm and among women 86.0 cm, which are lower than those reported at ^~^3600 MAMSL in La Paz-Bolivia (93 cm in women and 93 cm in men) ^35^ and close to those reported at ^~^3660 MAMSL in Tibet (84.5 cm overall) ^36^.

According to our results, by both WC and WtHR, Peruvians who live at higher altitudes have a lower prevalence of abdominal obesity than those living a lower altitude. This finding concurs with previous reports ^12, 37^; moreover, a higher percentage of overweight (36.3% vs 25.3%), obesity (17.5% vs 8.5%), hypercholesterolemia (18.9% vs 14.6%), low HDL (45.7% vs 40.3%), hypertension (9.8% vs 3.9%) and glycemia >126 mg/dL (2.9% vs 0.9%) were observed in people living above 3000 MAMSL vs below 1000 MAMSL ^37^. Overall, the lower cardiovascular risk observed at higher altitudes could be explained in part by the lower levels of urbanization and income, commonly reported in developing countries ^38^. Also, it might be explained by the variability in the progress of the epidemiological transition in Peru observed at different altitudes ^39^. It is important to highlight that a WtHR >0.5 seems to overestimate Peruvian abdominal obesity. Regardless of the evidence ^20^, if we use a cutoff of 0.5, over 80% of the Peruvian population is classified as having abdominal obesity. Further studies are needed to assess the usefulness of such an indicator in Latin-American countries such as Peru.

We should mention as a limitation that the ENAHO is a cross-sectional survey that was meant to represent Peru’s nutritional status, and the sample might not represent all altitudes of the country. Likewise, it is essential to emphasize that Peru is one of the few countries with many large populations over 3000 MAMSL. Therefore, the association between altitude and obesity could remain unnoticed at low altitude countries. Another limitation is the absence of variables such as socioeconomic status, education level, physical activity and diet. However, the area of residence (urban and rural) is a variable that encompasses socioeconomic and educational aspects in our country.

In conclusion, our study found that abdominal obesity is highly prevalent in Peru and that abdominal obesity varies substantially by altitude, age, gender, and urbanization. Overall, the prevalence of abdominal obesity decreases with altitude, but age and gender modify such association; abdominal obesity seems to affect older women from low altitudes more than younger men from high altitudes. These findings should help to guide interventions to reduce Peruvian’s cardiovascular risk, which should be a matter of more significant concern in future years.

## Data availability

### Underlying data

Figshare: Altitude and its inverse association with abdominal obesity in an Andean country. https://doi.org/10.6084/m9.figshare.9920234.v1 ^24^

This project contains the following underlying data:

- altitude_abdominal_obesity_dataset.xls (demographic and abdominal obesity data for participants)

Data are available under the terms of the Creative Commons Attribution 4.0 International license (CC-BY 4.0).
