# Supplementary material for: Altitude and its inverse association with abdominal obesity in an Andean country: a cross-sectional study
Source: F1000Res. 2019 Dec 27;8:1738. Originally published 2019 Oct 10. [Version 2] doi: 10.12688/f1000research.20707.2 (PMC6896245; doi:10.12688/f1000research.20707.2)
Supplement: Peer review history

# Peer review: Reviewer response for version 2

**DOI:** 10.5256/f1000research.24140.r58020

**Published:** 2020-01-07

**Authors:** Antonio Bernabe-Ortiz

**Affiliations:** 1 CRONICAS Center of Excellence in Chronic Diseases, Cayetano Heredia University, Lima, Peru

Although authors have improved the manuscript, they have not addressed all the comments submitted in the first review.

**MAJOR CONCERNS:**

**Methods:**

- If the ENAHO is conducted yearly, why to use the 2013 ENAHO? Why not to use the last one available? This needs to be explained or at least considered as a limitation.
- Was any criterion related to time living in high altitude (e.g. at least 6 months living in the city/area)? Are 30 days enough to see the potential impact of altitude on health? If not, please, add as limitation.
- How altitude was measured? Was GPS used for this? Only a simple calculation of the altitude of the city was used? Please explain. Usually, 2500 meters is used as the cutoff and not 3000... Since this variable is the main exposure, details should be given to understand if any misclassification could be introduced...
- Only those aged 20 years and more were included in the analysis? How about those between 18 to 20 years or those younger… why they were not included?
- Why education level and socioeconomic status were not confounders? As they are associated with obesity and people in high altitude settings, mainly rural, tend to have low SES and low education... should be they confounders? These variables are available in the ENAHO survey and must be included in analysis or demonstrate why not to include them?

 
**Results:**

- What do you mean for “703 different locations”? This part is not explained in the Methods section. Were they the areas with altitude information?
- Obesity prevalence must be standardized by age. Authors always said that the prevalence of obesity was lower in high altitude, but that is only the crude estimate... at least age standardization (if possible by sex also) must be conducted to say something like that.
- Please use appropriate graphs to see the inverted-u shape relationship... three points are not enough... use appropriate linear regression models? Or used more categories in the exposure variable... for example, an increase every 500 meters...

**Discussion:**

- What is the relevance of the findings? This is not clear as apparently the discussion is used only to compare results with other studies. A paragraph is needed to show the public health relevance of this association.
- I would also suggest to tone down some of the sentences as this is only a cross sectional study and not better analysis has been done to improve the understanding of the association between altitude and obesity prevalence.

 
**MINOR CONCERNS:**

- Change “Peruvian National Institute of Statistics and Information” for “Peruvian National Institute of Statistics and Informatics”.
- Please in Methods explain how what unreliable data defined.
- Usually there are no references in the Results section (verify #24).

I confirm that I have read this submission and believe that I have an appropriate level of expertise to state that I do not consider it to be of an acceptable scientific standard, for reasons outlined above.

# Peer review: Reviewer response for version 1

**DOI:** 10.5256/f1000research.22774.r56783

**Published:** 2019-12-05

**Authors:** Yuri Arnold Dominguez

**Affiliations:** 1 National Institute of Endocrinology (INEN), Havana, Cuba

In the topic number 1 and referring specifically to what was written in paragraph number one of the Introduction, I consider that not only the abdominal waist is the only predictor of abdominal obesity, there are other indicators as important as that, such as the Waist Index- size (taking into account that the Peruvian population prevails people with short stature), the waist waist index, the conicity index, which are usually more accurate than mere measurement than the abdominal circumference. I recommend that this investigation be continued in a larger sample , that it is even necessary to evaluate risk factors such as physical activity and diet, which are major confounders.

 Otherwise I catalogue the article as excellent.

I confirm that I have read this submission and believe that I have an appropriate level of expertise to confirm that it is of an acceptable scientific standard.

# Peer review: Reviewer response for version 1

**DOI:** 10.5256/f1000research.22774.r56784

**Published:** 2019-11-25

**Authors:** Ugo Fedeli

**Affiliations:** 1 Regional Epidemiology Service, Padua, Italy

The paper is interesting and well written. I have few suggestions:

 Methods, statistical analysis: please add some detail on interaction terms introduced in the log-binomial regression models; also in Results some interpretation about estimates obtained for the altitude x gender and altitude x age group would be useful for the reader.

 Page 6, second paragraph: it is stated that abdominal obesity and obesity prevalence were higher across rural areas than in urban areas; possibly the contrary should be reported.

 Table 5: my advice is to report in three separate columns estimates for unadjusted PR, adjusted PR without interaction terms, and adjusted PR with interaction terms (full model). It is not clear if PR reported in the Abstract are obtained from the full model; my advice is to report in the Abstract PR obtained by the adjusted model without interaction terms.

 In Discussion it is reported that differences in socio-economic status might be captured by the urban/rural variable. Are there also differences in ethnic background by altitude?

 Page 9, findings are summarized of a previous study reporting a higher percentage of overweight and other cardiovascular risk factors in people living above 3000 m vs. those living below 1000 m. However, these results seem to be in contrast with the present study; please explain.

I confirm that I have read this submission and believe that I have an appropriate level of expertise to confirm that it is of an acceptable scientific standard.

# Peer review: Reviewer response for version 1

**DOI:** 10.5256/f1000research.22774.r55852

**Published:** 2019-11-04

**Authors:** Antonio Bernabe-Ortiz

**Affiliations:** 1 CRONICAS Center of Excellence in Chronic Diseases, Cayetano Heredia University, Lima, Peru

***Overall comment:***

 This paper looks for the association between altitude and abdominal obesity. Although the idea is not novel, extra reports using existing data can help to understand better this. I would expect more details regarding the variables (exposure and outcome) used in the analysis. Moreover, any other kind of models, not that using only three categories for altitude would be more relevant, or verifying if the association is similar for overweight and obesity. This analysis can be improved by looking extra ways to see the association more than the traditional form to check that, for example, using linear regression if possible or polynomic models (quadratic at least).

***Major concerns:***

 Abstract:

 Please change it accordingly to comments below.

 Introduction:

- Obesity has different indicators and in the reference is used similarly for BMI (overall obesity), waist circumference (abdominal obesity), etc. Please be careful and consistent with words used.
- Some longitudinal studies have been published showing the association of interest and they have not been considered here (e.g. PMID: 29472520 ^1^).
- What is the novelty in this paper? Is it altitude? Apparently not as shown in the longitudinal paper... is it the rurality? Usually rural areas are in high altitudes. Is waist circumference an easy measure to do? Is it routinely done?
- How the usefulness of waist circumference in high altitudes is evaluated in this paper? I think that is no part of this study as pointed out in the last part of the first paragraph.
- Any reference for the last sentence of the second paragraph?
- Third paragraph: is the risk of obesity decreasing due to altitude? The reference is a cross sectional study, so is it possible to talk about risk?

Methods:

- Study design: why this study is multicentric? How many centers were included? Please explain.
- If the ENAHO is conducted yearly, why to use the 2013 ENAHO? Why not to use the last one available?
- Please explain how the sampling was done? Stratified by what? How many stages does the sampling have?
- Was any criterion related to time living in high altitude (e.g. at least 6 months living in the city/area)? Are 30 days enough to see the potential impact of altitude on health?
- Why households with 10 or more inhabitants were excluded? Any explanation? What proportion of households in Peru has 10 dwellers or more? Are these decisions biasing results?
- How altitude was measured? Was GPS used for this? Only a simple calculation of the altitude of the city was used? Please explain. Usually, 2500 meters is used as the cutoff and not 3000... Since this variable is the main exposure, details should be given to understand if any misclassification could be introduced...
- Only those aged 20 years and more were included in the analysis? How about those between 18 to 20 years or those younger?
- How the rural index was built? Explain please... Any reference helping to understand this? I am pretty sure the ENAHO stratifies the sample by urban/rural settings... was not the case this time?
- Categorization of BMI is presented in different way compared to how it was analyzed... please be consistent in definitions used
- “We assessed bivariate association using...” Association of what? Do you mean correlation between definitions of abdominal obesity? What was the gold standard?
- “Thus, we used a log-binomial...” Why education level or socioeconomic status were not confounders? As they are associated with obesity and people in high altitude settings, mainly rural, tend to have low SES and low education... should be they confounders? I know these variables are available in the ENAHO survey... why were they excluded?
- Ethic statement: where the dataset is available... add the link and how to get the data... I know a dataset has been added using Figshare, but that it not all the info available in the ENAHO dataset.

Results:

- What do you mean for “703 different locations”? This part is not explained in the Methods section. Were they the areas with altitude information?
- Education level and socioeconomic status should be included in the info as they are available in the ENAHO dataset.
- Waist to height ratio (definition) should be included since the methods section.
- Was obesity prevalence standardized by age? Authors always said that the prevalence of obesity was lower in high altitude, but that is only the crude estimate... at least age standardization (if possible by sex also) must be conducted to say something like that.
- “... we found that using the ATP III cutoff resulted in a stronger correlation with obesity”... what kind of obesity? Defined by BMI? By WC? By WtHr?
- Please use appropriate graphs to see the inverted-u shape relationship... three points are not enough... use appropriate linear regression models? Or used more categories in the exposure variable... for example, an increase every 500 meters...
- Would be good to know if stratification variables were effect modifiers? What was the test used for this? Although this was defined a priori, it would be good to have these estimates...

Discussion:

- I would suggest discussing about the relevance of this paper in the context of other papers (mainly those longitudinal).
- What is the relevance of the findings? This is not clear as apparently the discussion is used only to compare results with other studies. I would also suggest to tone down some of the sentences as this is only a cross sectional study and not better analysis has been done to improve the understanding of the association between altitude and obesity prevalence.

***Minor concerns:***

- Correct grammar spelling.
- Paper should be reviewed by an English native speaker.

I confirm that I have read this submission and believe that I have an appropriate level of expertise to state that I do not consider it to be of an acceptable scientific standard, for reasons outlined above.

## References

[1] : Trajectories of body mass index and waist circumference in four Peruvian settings at different level of urbanisation: the CRONICAS Cohort Study. *J Epidemiol Community Health*.72(5) : 10.1136/jech-2017-209795 397-403 10.1136/jech-2017-209795 29472520PMC5909748
